# Machine Learning Classifier-Based Metrics Can Evaluate the Efficiency of Separation Systems

**DOI:** 10.3390/e26070571

**Published:** 2024-06-30

**Authors:** Éva Kenyeres, Alex Kummer, János Abonyi

**Affiliations:** HUN-REN-PE Complex Systems Monitoring Research Group, Department of Process Engineering, University of Pannonia, Egyetem u. 10, P.O. Box 158, H-8200 Veszprém, Hungary

**Keywords:** waste sorting, Monte Carlo simulation, process development, classifiers, stochastic model

## Abstract

This paper highlights that metrics from the machine learning field (e.g., entropy and information gain) used to qualify a classifier model can be used to evaluate the effectiveness of separation systems. To evaluate the efficiency of separation systems and their operation units, entropy- and information gain-based metrics were developed. The receiver operating characteristic (ROC) curve is used to determine the optimal cut point in a separation system. The proposed metrics are verified by simulation experiments conducted on the stochastic model of a waste-sorting system.

## 1. Introduction

Separation processes have a wide range of industrial applications. The used raw materials often contain impurities that need to be removed before they are fed into the production system, and the products also need to be purified to reach the desired quality. Impurities often have to be removed from the raw materials, and the product needs to reach the appropriate purity. Our goal is to show that classifier-based metrics are suitable to represent the performance of separation systems regarding product quality and efficiency of the operation units. The introduction of these metrics into the evaluation of separation efficiency can help to achieve better product quality by optimizing the operating conditions, e.g., cut points.

Many types of separation tasks exist: the medium can be continuous (e.g., extraction [1]) or can consist of discrete parts (e.g., electrostatic separation [2] or size separation in a vibrated system [3]); the concept of separation can be based on mechanical principles (e.g., screening [4]), chemical (e.g., adsorption [5]) or thermal principles (e.g., distillation [6]); and the number of output streams can define binary or multicomponent separation [7]. All of these require the investment of energy or work to reach the desired product composition, which is different from the feed.

To evaluate and improve the efficiency of the separation systems described above, metrics are needed to quantify it. One approach focuses on the utilization of invested energy or work. In these cases, the efficiency metric is defined by the ratio of the useful energy and the invested energy, which is often mentioned as thermodynamic efficiency [6]. This efficiency metric has been used, e.g., for absorption, extraction and membrane separation processes in [1], adsorption processes in [5], and the optimization of cascade separation systems in [8].

Another approach takes into account the success of the separation regarding the separation goal that was defined relating to the expected product quality. In this case, the efficiency represents what percent of the component to be extracted in the feed is recovered. For example, a filtration or a freeze separation process can be characterized in this way [9,10]. Other efficiency metrics also consider the product purity besides the yield to represent the success of the separation. For example, in the case of solid–liquid separation, the amount of undesirable liquid in the product is also an important factor besides the recovery of the solids [11]. Newton efficiency (the product of the yield and improvement of quality) or Richarse’ efficiency (the product of the recovery for both the useful component in the product and the useless component in the residuum) are more appropriate metrics in this case [12]. The processes mentioned above all apply to binary separation, i.e., the system is divided into two distinct parts or media, and each of these product quality-based metrics characterize the system regarding the separation success of only one component.

When a multicomponent separation process is needed to be evaluated, one common metric including all information is more suitable since the multiple outputs of the system have to be assessed together to determine the separation efficiency, e.g., in case of a three-output distillation column [12]. In this case, a metric from information theory (e.g., entropy or information gain) can be an appropriate choice, which forms the third perspective regarding separation efficiency. Information gain derived from entropy is a metric originally used for the evaluation of classifier algorithms, e.g., decision trees [13]. As it characterizes the changes in the mixedness of a group of items, it was also successfully applied in the engineering field to evaluate the efficiency of a grinding process, where the mixedness was defined considering the distribution of the particle size [14]. Entropy-based metrics were also shown to be more suitable for evaluating separation systems than, e.g., Newton efficiency, as these have a high detection sensitivity in the whole range of product concentrations, and thus give a sensitive response even to small changes [15].

A separation process is similar to a classifier in the sense that it changes the mixedness of a medium, suggesting that other classifier-based metrics such as precision, accuracy, or recall in addition to entropy and information gain may also have a rational connotation in this interpretation. Moreover, receiver operating characteristic (ROC) curves, whose points represent the performance of the classifier under different classification thresholds [16], also seem applicable to evaluate the separation ability of a system, as they can function as a sensitivity analysis regarding the cut-off. ROC analysis is an efficient tool to visualize the performance of classifiers and compare them. It was used in signal detection theory and diagnostic systems first to illustrate the trade-off between hit rate and false alarm rate [17,18]. In the machine learning field, ROC curves were introduced by Spackman, who used them to assess model performance across different cut-off values and compare the performance of classifiers by using the Area Under the Curve (AUC) of the ROC curve as a metric [19]. This metric is often used in software engineering to assess binary classifiers; therefore, an extensive study about its reliability was also provided recently [20]. ROC curves have also been used to identify the cut-off value that results in the optimum trade-off between TP and FP values for a specific model fitting in a specific case study. For example, ROC curves have been used to adjust the balance between TP and FP in an early warning system for potentially dangerous hydrological events, thus enhancing its performance [21]. On the other hand, they are also often used to identify diagnostic biomarkers by comparing their ROC curves [22,23].

Despite the parallel between classifiers and separation systems, no work has been found exploring this issue or defining which other metrics from the machine learning field can be involved in the evaluation of industrial separation systems. Although machine learning models have been already used in separation systems, e.g., for the detection of components that need to be separated [24], or for the categorization of waste regarding recyclability [25], as another interesting linking point of the two fields, no work has been found on the interpretation of machine learning classifier-based metrics in an industrial separation system. Therefore, our work aims to investigate how classifier-based metrics can be used to evaluate product quality (e.g., purity and recovery) and the efficiency of operation units in the case of a separation process. As most of the practical examples mentioned above deal with fluids, our work focuses on systems where the medium to be processed consists of discrete parts.

In order to illustrate the applicability of classifier-based metrics to the evaluation of discrete separation processes, in this paper, a case study of a manual waste-sorting system is introduced. This type of sorting is still widely used and is also expected to be used in the future for preselection before mechanical sorting equipment to enhance its efficiency or to remove certain contaminants (e.g., dirty or oily items) [26]. Manual sorting is also preferable in the case of parts with large weights which implies a lower efficiency of mechanical sorting, or in the case of valuable fractions [27]. Although robotics solutions are also spreading in this field, their high investment cost creates a barrier that preserves the need for manual waste-sorting systems [28].

In general manual sorting systems, waste is transported on a conveyor belt with operators on both sides [26]. In our case study, the conveyor belt is considered to be divided into parallel zones; the number of zones corresponds to the number of defined classes referring to different product types. The operators can move the trash pieces between these zones (or even take them off from the conveyor belt), thus changing the distribution of the waste; thereby, multicomponent separation takes place. Here, operators can be considered “operation units”, whose effect on the waste distribution can be evaluated by the classifier-based metrics from the machine learning field described above.

The operation of this system is dynamic, and some random phenomena also appear, for instance, the precision of the operator actions, the material quality, and the arrival time of the new trash pieces, which is modeled involving queuing theory [29]. Therefore, a stochastic model is needed to describe the system, which can be solved by Monte Carlo simulation to achieve robust results, focusing on the mean and confidence intervals of the previously defined metrics.

In terms of analyzing the waste-sorting efficiency, most of the related literature applies purity-based metrics. Grade and recovery are used to determine the optimal operating conditions of an electrostatic separation process for plastic waste [30]. The separation distance proportional to the purity of the product is applied to improve the efficiency of an eddy current separator [31]. A weighted average purity metric is defined based on the concentration of the output streams to compare the configurations of the multistage separation lines [32]. However, entropy-based metrics are only used to qualify the separation complexity of mixed waste but not for the evaluation of a waste-sorting system [33]. The authors aim to fill this gap by choosing a case study related to waste separation.

The aim of this paper is to define metrics for the evaluation of multicomponent separation systems, which can be easily interpreted, visualized, and applicable for process development purposes. The metrics used for the assessment of classifiers are applied to reach these goals and qualify the operation of the separation system and product quality. Some of them (e.g., ROC curve) can also help to ensure high operating performance by optimizing the parameters of the system. As an example, the operational analysis of a manual waste-sorting process embodying a discrete separation system is introduced, and its performance is evaluated with respect to operator efficiency and the quality of sorted output waste streams.

The contributions of the paper are the following:

- A concept for the evaluation of multicomponent separation system performance using classifier-based metrics;
- Description of the stochastic model of the improved waste-sorting system;
- Investigations on how the entropy and information gain characterize the “operation unit” efficiency;
- A technique for using the ROC curve to find the optimal value of the separation threshold parameter of the system.

The roadmap of the paper is the following: in Section 2, the methodological background of the study is described. Section 2.1 reveals the connection between classifiers and separation systems; the connotation of traditional classifier-based metrics in a separation system is defined in Section 2.2; and Section 2.3 extends the interpretation of metrics related to binary systems for multicomponent ones. Section 2.3.1 contains ideas on the application of ROC curves in a separation system, and Section 2.3.2 reveals the connection between traditional efficiency metrics of separation systems and the classifier-based ones. In Section 3, a case study of a manual waste-sorting system is provided to support the ideas above. Section 3.1 introduces the framework of the model that was created to describe the system with stochastic phenomena; Section 3.2 focuses on the implementation of classifier-based metrics to evaluate the system; Section 3.3 and Section 3.4 introduce the experimental setup and the results, respectively. In Section 3.5, a method is proposed to apply the ROC curve to determine optimal separation thresholds. Finally, Section 4 summarizes the main conclusions together with the limitations of the proposed method, and reveals some future research directions as well.

## 2. Application of Classifier-Based Metrics in Separation Systems

Separation systems require performance metrics that are easily interpretable and applicable to achieve process development aims. As a separation task is basically a classification problem, the metrics used for the evaluation of classifier algorithms can be involved in defining separation efficiency regarding product quality and operation unit efficiency. This work focuses on discrete separation systems primarily; however, the introduced concept is similarly applicable to continuous flows, with minor modifications.

In Section 2.1, a discrete separation problem is formalized under the scope of the classifiers, and Section 2.2 reveals the meaning of classifier-based metrics in the case of a separation process. Section 2.3 introduces how the metrics originally for binary systems (e.g., ROC curve) can be interpreted in a multicomponent case and makes a connection between the traditional and classifier-based ones.

### 2.1. Interpretation of Separation Problems as Classifier Systems

The aim of a separation process is to divide the input stream into two (binary separation) or more (multicomponent separation) output streams with different compositions from the feed and each other. Each component to be recovered is expected to accumulate in one of the output streams as much as possible. Thereby, each output stream is enriched in some of the components and impoverished in others.

The factor that the separation is based on can be, for example, in discrete systems the shape, color, or material quality of the items. These items are reordered by location such that they leave the system in the right output stream. The aim is to gain the purest output streams as possible with the least energy or work invested.

**Figure 1:**
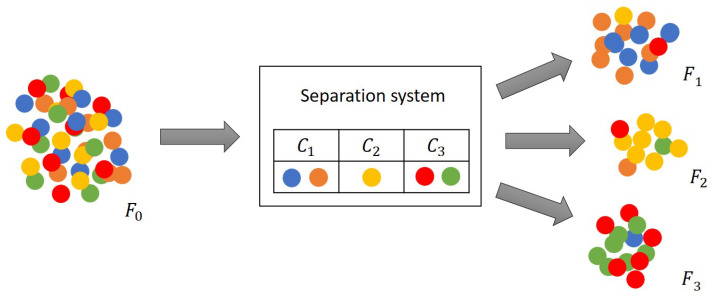
Schematic figure of a general separation system with three output streams, i.e., three target classes. The input ($F_{0}$[pcs]) and output streams ($F_{j}\;\left[pcs\right]\in \left\{F_{1},F_{2},F_{3}\right\}$) are shown, and the separation task defined by $C_{i}\in \left\{C_{1},C_{2},C_{3}\right\}$ class labels. The hypothetical components are marked by different colors.

The essence of a classification problem is quite similar: ordering $N_{S}$ sample elements or objects into $N_{C}$ groups called classes according to a specific feature which distinguishes them [16]. A general separation task can be considered in this manner as shown in Figure 1 schematically.

The system feed ($F_{0}$) contains objects of many components mixed, and the goal is to sort them into predefined classes ($C_{1},...,C_{N_{C}}$) to leave the system in different output streams ($F_{1},...,F_{N_{C}}$). Figure 1 illustrates a batch separation process; however, a continuous one can be formalized similarly except that the dimension of the streams is $\frac{pcs}{time}$.

The classes are created by grouping the components as seen in Figure 1. For example, the objects of the red and green components are collected together in class $C_{3}$, such as those of the blue and orange components in class $C_{1}$. Considering continuous mediums as input and outputs, this scheme is appropriate to describe the fractional distillation of crude oil, where the cut point temperatures determine which chemical species leave the column in the same output stream [34].

The final purpose of a separation system is also similar to that of the classification problems: obtaining the purest groups or output flows as possible, i.e., assigning to the appropriate class labels as many pieces as possible. However, in practical problems, completely pure outputs are rarely formed. As can be seen in Figure 1, the majority of the elements are in the right output (or class); however, there are some impurities in all product streams. Therefore, the quality of the products must be assessed, which is presented in Section 2.2.

### 2.2. Classifier-Based Metrics for the Evaluation of Multicomponent Separation

**Figure 2:**
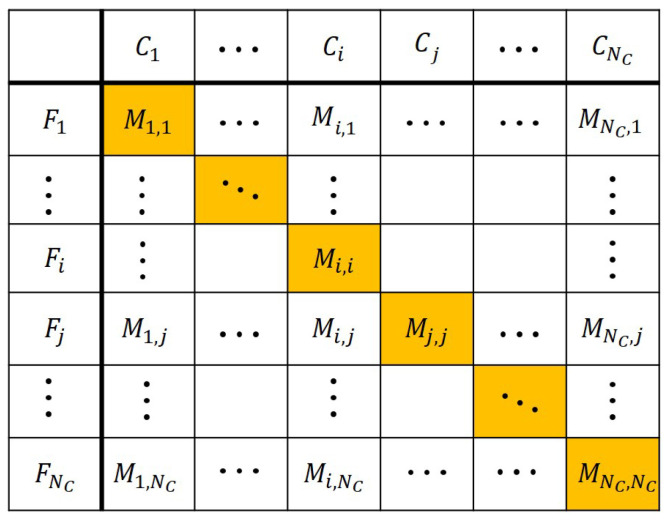
Confusion matrix of a general separation system. $M_{i,j}\;\left[pcs\right]$ refers to the number of the elements that belong to the $C_{i}$ class in the $F_{j}$ stream.

Obviously, a separation process rarely results in completely pure output streams as is also shown in Figure 1. The above-mentioned analogy allows to utilize the same metrics for the evaluation of the efficiency of a discrete separation system as in the case of a general classifier model. The confusion matrix can be created to assess the sorting efficiency, showing the distribution of class components among the gained groups or the output flows as seen in Figure 2 [16].

In this interpretation, the “actual value” is the label of the $C_{i}$ class, to which the given sample element belongs, and the “predicted value” is the output stream $F_{j}$ that contains it. $M_{i,j}\;\left[pcs\right]$ counts the elements in the $F_{j}$ stream that belong to the $C_{i}$ class. The sample elements that are correctly placed (i.e., the elements of class $C_{i}$ that are in the $F_{i}$ output stream in which the elements of that class are expected to accumulate) are accounted for in the orange cells in Figure 2.

In the special case of two-class classification, the classes are often labeled as *positive* and *negative*, and the four cells of the confusion matrix are designated as *true positive* (TP), *false positive* (FP), *true negative* (TN) and *false negative* (FN) [16]. In case of a general multiclass separation problem for which the confusion matrix is formalized in Figure 2, these terms can be defined as the following: considering a certain class ($C_{i}$), true positive ($TP_{i}$) refers to the elements of class $C_{i}$ that are in the right output stream ($F_{i}$), and false positive ($FP_{i}$) collects all the other elements of that stream. False negative ($FN_{i}$) denotes the elements of the $C_{i}$ class that are in wrong output streams (other than $F_{i}$), and true negative ($TN_{i}$) denotes the correctly placed elements of all the other classes. In the confusion matrix of Figure 2, the orange cell in column $C_{i}$ belongs to $TP_{i}$, and the sum of all other (white) cells in the column to $FN_{i}$. $FP_{i}$ is considered the sum of white cells in the row of $F_{i}$, and $TN_{i}$ includes all orange cells on the main diagonal except the one currently labeled as $TP_{i}$.

Relying on these basic terms, other classification quality metrics such as *precision*, *recall* and *accuracy* can be defined, which all have a reasonable meaning in terms of a separation system, as well. Precision actually refers to the purity of the output streams here. It is the ratio of correctly classified elements in the $F_{j}$ stream and can be calculated as:

(1) $$Precision_{j}\;\left[\%\right]=\frac{{TP}_{j}}{{TP}_{j}+{FP}_{j}}=\frac{M_{j,j}}{\sum_{i=1}^{N_{C}}M_{i,j}}\cdot 100\%=p_{j,j}\cdot 100\%=Purity_{j}\;\left[\%\right],$$

where $p_{j,j}$ refers to the ratio of the elements in $F_{j}$ that belong to the *j*th class.

The recall metric corresponds to the recovery in a separation system. It expresses what percent of the elements of a class have been extracted in the desired output stream. Recall can be computed as:

(2) $$Recall_{i}\;\left[\%\right]=\frac{{TP}_{i}}{{TP}_{i}+{FN}_{i}}=\frac{M_{i,i}}{\sum_{j=1}^{N_{C}}M_{i,j}}\cdot 100\%=Recovery_{i}\;\left[\%\right].$$

As can be seen in Equations (1) and (2), precision (purity) characterizes a stream, and recall (recovery) is related to class, i.e., set of components desired to take together in the same stream. The accuracy metric, however, characterizes the performance of the whole system by defining the ratio of elements placed correctly (from all classes) in relation to all elements:

(3) $$Accuracy\;\left[\%\right]=\frac{{TP}_{j}+{TN}_{j}}{{TP}_{j}+{TN}_{j}+{FP}_{j}+{FN}_{j}}=\frac{\sum_{j=1}^{N_{C}}M_{j,j}}{\sum_{j=1}^{N_{C}}\sum_{i=1}^{N_{C}}M_{i,j}}\cdot 100\%.$$

*Information entropy* describes the heterogeneity of the mixture, which is expected to decrease during the separation process. As such, it is particularly suitable to evaluate the efficiency of the separation system considering the information gain caused by it. The information entropy can be calculated using its defining equation and complementing it with a normalization factor in the denominator to ensure that its value remains between 0 and 1 independently from the number of classes as was suggested for multicomponent cases in [35]:

(4) $$H_{j}=\frac{\sum_{i=1}^{N_{C}}-p_{i,j}\log_{2}p_{i,j}}{\log_{2}N_{C}}=\frac{\sum_{i=1}^{N_{C}}-\frac{M_{i,j}}{\sum_{i=1}^{N_{C}}M_{i,j}}\log_{2}\left(\frac{M_{i,j}}{\sum_{i=1}^{N_{C}}M_{i,j}}\right)}{\log_{2}N_{C}},$$

where $H_{j}$ refers to the entropy in stream $F_{j}$, and $p_{i,j}$, which refers to probabilities in the traditional perspective of information entropy, represents the ratio of the elements in $F_{j}$ that belong to the $C_{i}$ class here.

If the entropy of the output streams altogether is wanted to be determined to characterize the overall performance of the separation system, the $H_{j}$ individual entropy measures of streams can be weighted ($w_{j}$) according to the number of sample elements in the streams $F_{j}$, and the full information content of the medium representing the separation complexity or mixedness can be calculated as:

(5) $$H=\sum_{j=1}^{N_{C}}H_{j}w_{j}=\sum_{j=1}^{N_{C}}H_{j}\frac{\sum_{i=1}^{N_{C}}M_{i,j}}{\sum_{j=1}^{N_{C}}\sum_{i=1}^{N_{C}}M_{i,j}},$$

which also gives information about the *information gain* (IG) during the separation process as:

$$IG=H_{0}-H,$$

where $H_{0}$ represents the initial entropy of the waste.

The classifier-based metrics introduced above together with their associated meanings for a separation scheme are collected in Table 1.

As can be seen in Table 1, precision and entropy ($H_{j}$) both characterize the $F_{j}$ streams. However, entropy holds much more information since it considers not only the ratio of the correctly placed elements belonging to the $C_{j}$ class in $F_{j}$ like the precision metric but also the distribution of the elements that were incorrectly placed in the $F_{j}$ stream. As seen in Equation (4), the formula of $H_{j}$ contains all ${\left\{p_{i,j}\right\}}_{i=1}^{N_{C}}$ values, in contrast to the precision, which only deals with $p_{j,j}$. The case is similar when comparing accuracy, overall entropy, and information gain: all of them characterize the performance of the whole separation system, i.e., the output streams altogether in one metric. However, accuracy only gives the overall ratio of the correctly placed elements, while *H* (and IG) carries information about the complexity of the output streams (and the change of it) as well.

**Figure 3:**
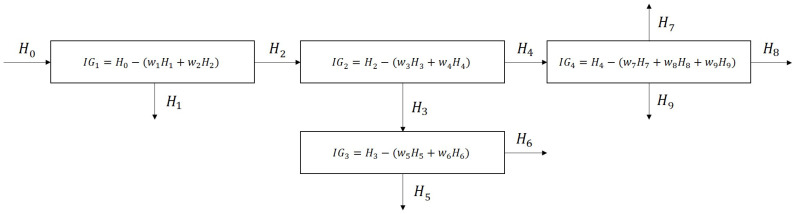
Evaluation of the individual operation units of a separation system by information gain ($IG_{i}$) computed using entropies ($H_{j}$) of their input and output streams. The entropies are weighted according to the ratio of the output streams of the units, thereby, $w_{1}+w_{2}=w_{3}+w_{4}=w_{5}+w_{6}=w_{7}+w_{8}+w_{9}=1$.

From all the metrics in Table 1, IG is the only one which is relative and clearly reflects the effect of the separation system on the medium. Thereby, in case of a multistage separation system, the IG metric is also applicable to quantify the effect of the operation units on the mixedness of the medium individually as can be seen in Figure 3, considering the input and output streams of the appropriate unit.

This metric will be used in our waste-sorting case study that will be introduced in Section 3 to evaluate and visualize the effect of the operators (considering them “operation units”) on the separation complexity of the waste.

### 2.3. Metrics for Binary Systems in a Multicomponent Case

In case of a binary separation process, two output streams are formed, i.e., two classes are defined. It is often used, for example, for the separation of a valuable component from a useless one. The former is expected to concentrate in the product stream and the latter in the stream called residuum. Multicomponent separation can also be considered this way, if only one valuable component is in the focus. In this case, the *specificity* metric of classifiers has also a rational meaning, namely, the recovery rate of the useless component in the residuum:

$$Specificity=\frac{TN}{TN+FP}.$$

On the other hand, ROC curves can be also applied for evaluating multicomponent systems, and traditional efficiency metrics interpreted by classifier-based metrics based on this perspective, which will be introduced in this section.

#### 2.3.1. ROC Curve in a Separation System

The receiver operating characteristic (ROC) curves graphically represent the quality of binary classifiers originally. This tool gives the chance to investigate sensitivity and specificity metrics together, thus providing a clearer picture about the performance of the classifier, and also applicable for separation systems, consequently.

The points of the curve are obtained by performing the classification with different classification thresholds. In each case, the *true positive rate* ($TP_{rate}$) is determined and illustrated by the related *false positive rate* ($FP_{rate}$). $TP_{rate}$ is equal to the recall metric, and $FP_{rate}$ is calculated as [16]:

$$FP_{rate}=\frac{FP}{TN+FP}=1-Specificity.$$

The classification threshold in a separation system corresponds to a characteristic parameter of the system, which defines the cut-off point. Although the ROC curve is for binary classifiers/separation systems originally, it can be drawn in the multicomponent case as well, by focusing only on one of the separation thresholds. Thereby, ROC curves can even be created for each cut point and investigated together.

The Area Under the ROC Curve (AUC) refers to the quality of the classifier, i.e., the separation system. As both $TP_{rate}$ and $FP_{rate}$ take values between zero and one, the maximum of AUC is one. The larger the AUC, i.e., the more the ROC curve approximates the (0,1) point in the graph, the more efficient the classifier.

#### 2.3.2. Connection between Traditional and Classifier-Based Metrics for Separation Efficiency

Considering the classifier-based metrics mentioned above, their connection with Newton and Richarse’ efficiency can be formulated as follows. These were also used for binary separation originally; therefore, the efficiency of the separation regarding one component or component class can be characterized by them in a multicomponent system.

The Newton efficiency ($\eta_{N}$), which is the difference of the recovery of the valuable component (or component class) and the intermix rate of the useless component into product, can be defined as:

(6) $$\eta_{N}=Recall-\left(1-Specificity\right).$$

It can be seen that the two members of Equation (6) correspond to the metrics on the two axes of the ROC curve. Therefore, Newton efficiency can be written as:

$$\eta_{N}=TP_{rate}-FP_{rate},$$

and can be calculated from the ROC curve for different separation thresholds.

The Richarse’ efficiency ($\eta_{R}$), which is the product of the recovery of the valuable component in the product and the recovery of the useless component in the residuum can be written as:

$$\eta_{R}=Recall\times Specificity.$$

These definitions ensure the interoperability between traditional metrics and classifier-based metrics. In Section 3, a short analysis of the ROC curve and Newton efficiency gained from it will also be presented.

## 3. Applications in the Evaluation of a Waste-Sorting System

A manual waste-sorting system is introduced here as an illustrative example of the previously defined discrete separation systems. These days, it is essential to have well-separated waste fractions to achieve sustainability goals such as incrementation of the recycling performance or the reduction of pollution [28]. Manual waste separation is still a widely used solution due to lack of expensive equipment. In this section, a new concept based on stochastic discrete simulation is presented to increase the separation efficiency. It can be even used for preselection tasks to improve the performance of available equipment if there is one.

In manual waste separation systems, operators perform the sorting. They can be considered as operation units, whose effect on waste distribution is evaluated using classifier-based metrics in this section.

### 3.1. Concept of the Waste-Sorting System

**Figure 4:**
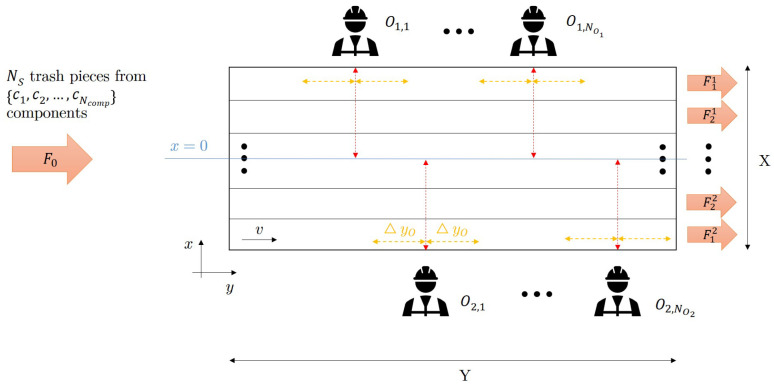
Schematic figure of the manual waste separation process. Operators on the one and the other side of the conveyor are denoted by $O_{1,m}$ and $O_{2,n}$, and the output stream by ${\left\{F_{i}^{1}\;\left[pcs/s\right]\right\}}_{i=1}^{N_{C}}$ and ${\left\{F_{i}^{2}\;\left[pcs/s\right]\right\}}_{i=1}^{N_{C}}$, respectively. The feed ($F_{0}\;\left[pcs/s\right]$) contains $N_{comp}$ type of components marked by ${\left\{c_{l}\right\}}_{l=1}^{N_{comp}}$. The operators can reach a distance of $\Delta y_{O}$ in the *y* direction.

The waste separation system consists of a conveyor belt and a certain number ($N_{O_{1}}$ and $N_{O_{2}}$) of operators on the two sides of it as can be seen in Figure 4. The trash pieces arrive at the conveyor belt from a vibration plate which aims to distribute the pieces evenly. The trash pieces move with a constant *v* speed on the conveyor belt. The sorting efficiency can be increased if the operators not only take off pieces from the conveyor but also rearrange the waste there. A theoretical model of this system is introduced here, where the stochastically arriving pieces have to be classified dynamically.

As can be seen in Figure 4, the conveyor belt is divided into zones belonging to the component classes that need to be separated. The zones are labeled axially symmetrical to the x=0 line, as it is assumed that the operators can reach only the middle of the conveyor belt regarding the *x* direction.

The input and output streams are marked similarly as before, except that their dimension is $\frac{\mathrm{pcs}}{\mathrm{time}}$ here because of the dynamic nature of the system. Thereby, the $M_{i,j}$ values of Equations (1)–(5) can be considered integrals over time as will be defined in Section 3.2. The $F_{0}$ feed contains $N_{S}$ trash pieces being from one of $\left\{c_{1},c_{2},...,c_{N_{comp}}\right\}$ components which are grouped into predefined $\left\{C_{1},C_{2},...,C_{N_{C}}\right\}$ classes according to the separation aims. The output streams have a superscript in addition, which refers to the half of the conveyor belt to which they belong. Thereby, $F_{i}^{j}$ denotes the zone where the components of the $C_{i}$ class are collected under the authority of the ${\left\{O_{j,m}\right\}}_{m=1}^{N_{O_{j}}}$ operators and the number of output streams is $2N_{C}$. The operators reach over half of the conveyor belt in the *x*, and a distance of $\Delta y_{O}$ in the *y* direction.

The model of the system setup uses the Lagrangian approach borrowed from field theory, where it supports the representation of fluid flow by tracking the motion of individual fluid particles [36]. Thereby, here, the trash pieces are tracked and their paths followed. Their position on the belt in two dimensions and their material quality are assigned to them.

The operators can be considered consecutive “operation units” that change the *x* position of the trash pieces, i.e., the distribution of the waste on the conveyor. To ensure the most efficient operation, they always choose the last trash piece in a wrong zone that they reach (i.e., the one with the highest *y* position in their authority) to move. Operator actions are considered a stochastic process in the model, and their inaccuracy can be characterized by σ, which represents the uncertainty of placing the trash piece in the middle of the target zone. If its value is high, it can even happen that the moved trash piece arrives in the wrong zone as is explained in more detail in Appendix A.2, together with other stochastic phenomena in the system.

**Figure 5:**
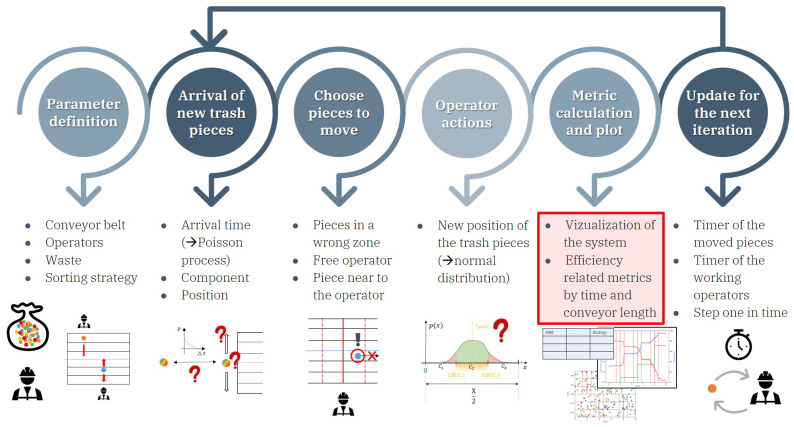
Graphical abstract of the created simulator of the manual waste-sorting system.

The graphical abstract of the simulation framework created is shown in Figure 5. It can be considered a discrete event simulator (DES), as the trash pieces remain undisturbed between their birth and the operator actions.

As a first step, the user has to define the parameters such as the size of the conveyor belt, the number and location of the operators together with their action time and precision, the waste load and composition, and the sorting strategy, i.e., which components are wanted to be taken off from the belt, and which ones are wanted to be collected together in the same zone of the belt. The model is dynamic and is discretized by time; thereby, the next five steps are repeated iteratively every time step. The arrival of the new trash pieces has to be defined first. The birth of them is modeled as a Poisson process; thereby, the interarrival times follow an exponential distribution. The component type of the new trash pieces are chosen randomly based on the composition of the feed, and the items arrive at every position along the width of the conveyor with equal probability. Then, new pieces to move are chosen for the free operators, and the operator actions are executed as described above. The action time is handled by timers in the case of the moved pieces and the operators as well, which are checked before the next iteration step. If any of them are over the action time, the related piece or the operator becomes free. At the end, performance metrics that will be introduced in Section 3.2 are also calculated, and the system is visualized dynamically.

The detailed model equations of the system with some additional considerations are introduced in Appendix A.

### 3.2. Performance-Related Metrics

**Figure 6:**
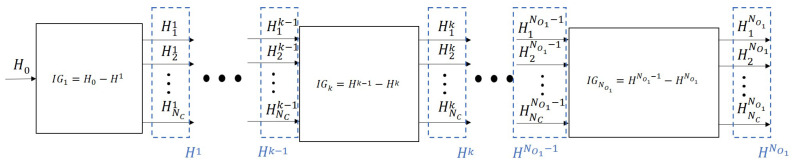
Representation of the manual waste-sorting system as consecutive multi-input multi-output units, and their evaluation by information gain (IG). $H_{0}$ refers to the entropy of the feed, $H_{j}^{k}$ to the *j*th output stream of the *k*th operation unit, and $H^{k}$ denotes the overall entropy of the *k*th unit. $N_{O_{1}}$ marks the number of operation units representing the operators at the x>0 side of the conveyor.

Since operators have limited working capacity, the waste probably will not be completely separated while the conveyor passes the first operator (of course, it depends on the speed of the conveyor and load, too). Therefore, multiple operators are usually employed on both sides of the conveyor belt. They can be considered identical operation units, consecutively, that the waste streams flow in and out of. In this case, all the semi-sorted streams leave the first unit, reach the second one, where they become more pure, and so on. Thereby, Figure 3 can be rearranged as shown in Figure 6.

As the system is symmetric to the line x=0, and the two halves are completely independent, as the operators can reach until the center line, only the x>0 half of the conveyor belt is considered in Figure 6 and in the following part of the paper. The performance of the operation units representing the operators can be evaluated by analyzing the output streams of the units using the classifier-based metrics (precision, entropy, information gain, etc.) introduced above. The aim is to identify changes in the distribution of the waste due to the operator actions along the *y* axis.

The precision metric is applicable here to represent the purity of the waste stream in a unique zone. It is a crucial factor in case of the component classes, where the aim is to remove impurities as much as possible, e.g., components that are further processed or recycled. Recall can be useful for component classes, where it is more important that all the class elements are gathered in a unique stream than preserving the purity of that stream. For example, the removal of contaminated waste from the reprocessable streams can be characterized by this metric. Accuracy defines the overall ratio of the pieces in the right zones. Entropy represents the complexity or mixedness of the individual ($H_{j}$) or the total waste streams (*H*). The latter also defines the information gain (IG), considered here the effect of the operators.

The system is dynamic, and the waste consists of discrete pieces; thereby, the specified metrics can be evaluated by taking the average along the *y* axis. For each $y_{i}$ value of the *y* axis, the set of the last $N_{p}$ trash pieces that passed through the $y=y_{i}$ line was identified, their data collected, and their confusion matrix created. The specified metrics assigned to $y=y_{i}$ can be calculated based on it. In our simulation experiments, $N_{p}=50$ was used.

### 3.3. Experimental Setup

To show how classifier-based metrics are appropriate for analyzing the performance of separation systems, simulation experiments were performed on the manual waste-sorting system described above.

The experimental setup was the following. The conveyor belt was considered 1 m wide (*X*) and 10 m long (*Y*) moving with $0.1\;\frac{m}{s}$ constant speed. The trash pieces were assumed to arrive at $4\;\frac{\mathrm{pcs}}{s}$ speed at the beginning of the conveyor. All operators were considered to reach 0.7 m along the *y* direction ($\Delta y_{O}$) with 2s action time. Three of them were applied standing consecutively on the side of the belt 2, 5 and 8 m from the beginning of the conveyor. The composition of the waste feed was assumed as shown in Table 2.

**Figure 7:**
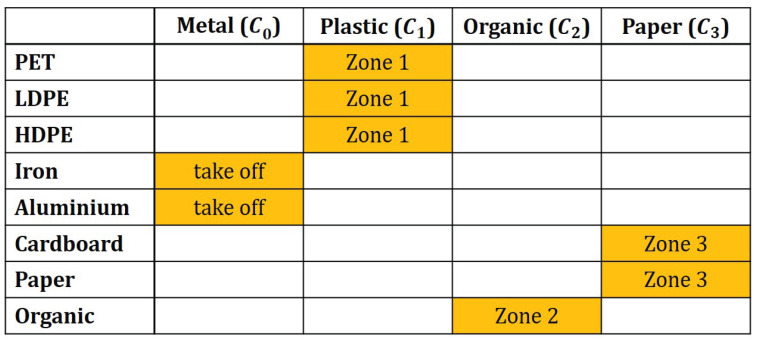
Projection between the components in the first column and classes in the first row of the table. In each column, the components that belong to the actual class are marked by orange, and it is also mentioned where they are collected.

Four classes are formed from these components: plastic (15.6%), metal (3.7%), paper and cardboard (36.4%), and organic (44.3%) fractions should be separated. Metal is collected by taking it off from the conveyor belt, and the other three fractions are separated by rearranging the elements on the conveyor belt. Thereby, three zones are defined by dividing the belt equidistantly. The projection matrix of the components for the classes is shown in Figure 7.

The simulation was run long enough to bring the system to a steady state. Due to the stochastic nature of the process, Monte Carlo (MC) simulation was used; the results introduced in Section 3.4 are the summarization of 20 independent runs.

### 3.4. Experimental Results

In this section, the evolution of the previously defined classifier-based metrics along the conveyor belt is introduced. The following figures summarize the results of 20 independent runs of the experiment setup described above; the mean and the quartiles are illustrated. The positions of the operators are marked by vertical red continuous lines, and the territory that they can reach over is bordered by dashed lines.

**Figure 8:**
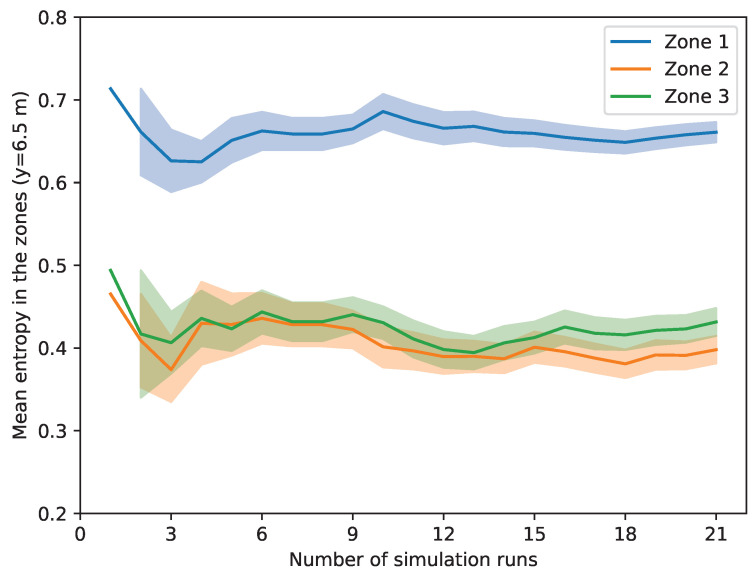
An example of convergence analysis of the entropy in the zones at y=6.5 m. *y* refers to the length dimension of the conveyor as can be seen in Figure 4.

The number of simulation runs was determined by analyzing the convergence of the mean values [37]. We performed investigations at different points of the conveyor length for all metrics. As an example, Figure 8 illustrates the mean values of entropy in the zones together with the quartiles along an increasing number of simulation runs at the y=6.5 m point of the conveyor length, representatively.

It can be seen in Figure 8 that the mean values are nearly constant above 15 simulation runs; therefore, 20 runs seem appropriate for the investigations. The investigated metrics are related to a given amount of trash pieces, and calculated by taking the average as declared in Section 3.2. This fact explains the relatively low number of required runs.

**Figure 9:**
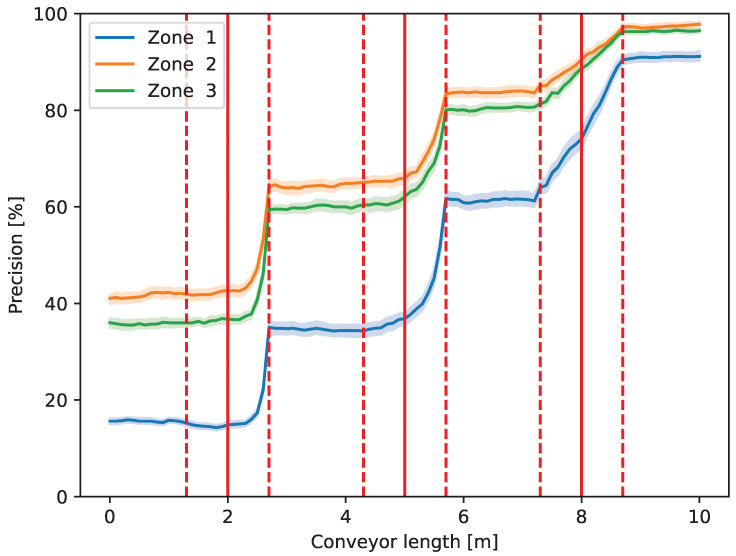
Precision [%] representing purity in the zones along the conveyor length (*y* axis in Figure 4).

In Figure 9, the precision metric related to the purity of the flow in the zones is shown.

As can be seen in Figure 9, the precision that represents the purity of the component collected in the actual zone monotonically increases due to the operator actions in all zones. The initial value of the precision corresponds to the composition of the feed. After the third operator, precision approaches 100% in all zones, which means that the output flows are close to pure materials besides the defined parameters.

**Figure 10:**
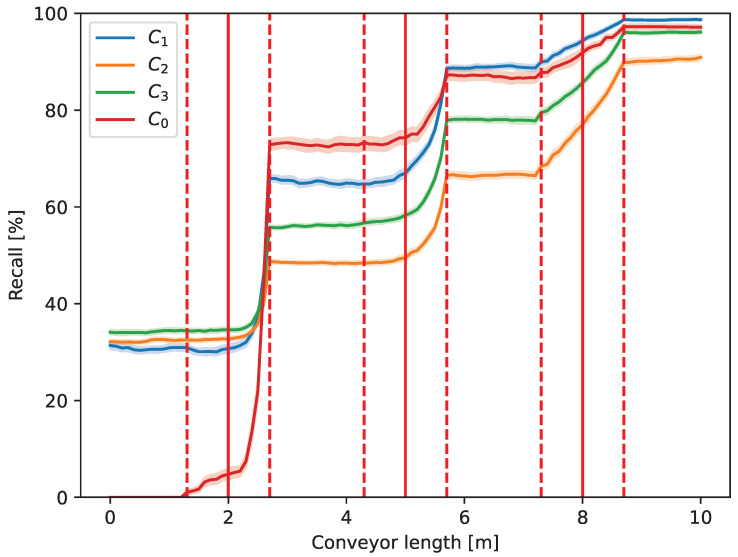
Recall [%] of the component classes along the conveyor length (*y* axis in Figure 4).

Figure 10 illustrates the recall metric that represents the recovery of the component classes.

The recall of the $C_{0}$ class that was collected by taking the elements off from the conveyor belt is also reasonable to analyze and, thereby, is illustrated in Figure 10 as well. The recall, similarly to the precision, also increases monotonically by the effect of the operators. The initial values are equal in the zones, as the trash pieces are randomly distributed at the beginning of the belt. However, elements of the $C_{0}$ class to be taken off are all on the conveyor initially; thereby, its recall is zero at the beginning of the belt. It can also be seen that the higher the concentration of the component class in the system, the less its recall increases.

**Figure 11:**
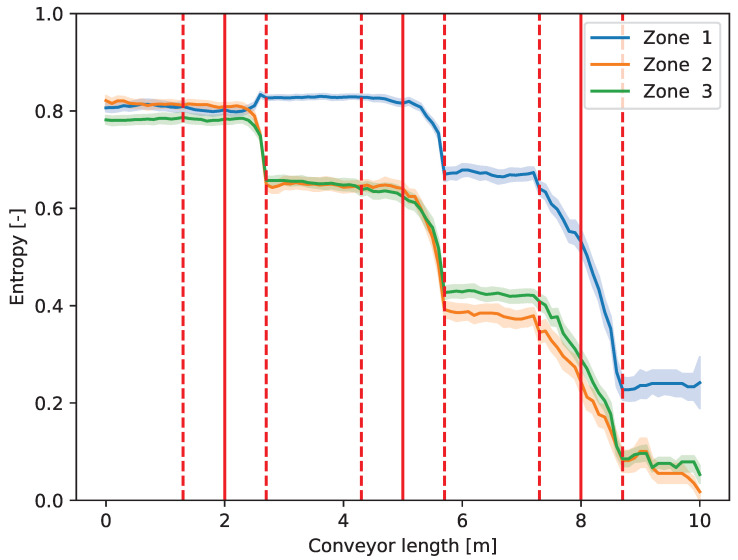
Changes in the entropy of the zones ($H_{j}$) along the conveyor length (*y* axis in Figure 4).

Figure 11 illustrates the $H_{j}$ entropies of the zones. It can be seen in Figure 11, that in Zone 2 and Zone 3, the entropy decreases monotonically by the work of the operators; however, in Zone 1, where plastic is collected, giving only 15% of the waste, the entropy increases by the effect of the first operator and begins to decrease only after the second one. The reason for this is the asymmetric waste composition. The entropy is maximal when the ratio of every class is equal. In our experiment, as the operators begin to move the wrong pieces, which are present in high concentration (organic and paper fractions), from Zone 1 to Zone 2 and Zone 3, the composition of Zone 1 starts to even out at the beginning (entropy increases) until the system is beyond a balanced state. Thereby, entropy in Zone 1 reaches a maximum, and the composition tends to become imbalanced only after that, in favor of the component concerned at this time.

**Figure 12:**
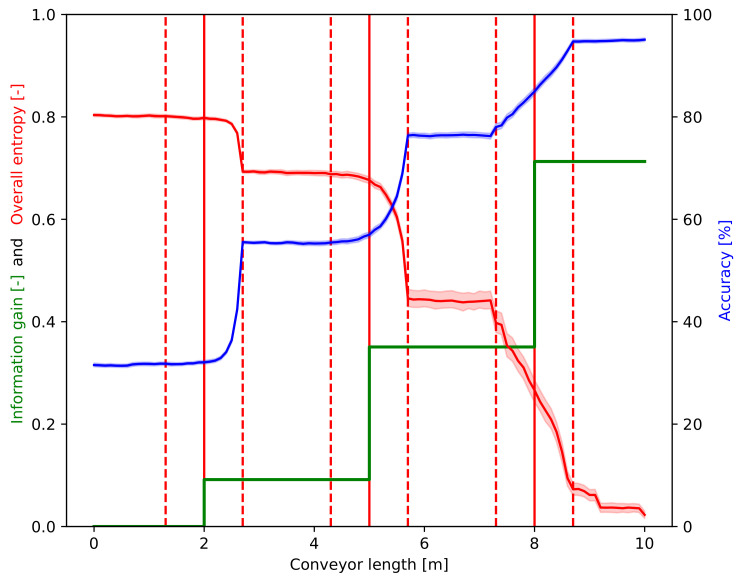
Metrics characterizing the whole system in one: accuracy [%], overall entropy (*H* [-]), and cumulative information gain (IG [-]) along the conveyor length (*y* axis in Figure 4).

Figure 12 represents the evolution of the accuracy, overall entropy, and cumulative information gain metrics along the conveyor belt. Figure 12 illustrates the three metrics that were declared to characterize the whole system in Table 1 for comparison. Accuracy is presented in percent on the right y-axis, and values of *H* and IG are measured in the left y-axis and appear between 0 and 1. The cumulative information gain curve is intended to indicate the overall effect of the operators before *y*, and the change that is caused by them are assigned to the position of the operators.

As the waste passes in front of more and more operators, the accuracy, i.e., the ratio of the elements in the right zone, is increased, and the overall entropy is decreased. On the other hand, the effect of the defined sorting strategy also appears in Figure 9, Figure 10, Figure 11 and Figure 12. Always, the element farthest forward on the conveyor within the authority of the operator is chosen to move. As the operators work slower (higher action time) than the new elements to be moved arrive at the conveyor, they are overwhelmed, and the element to be chosen is close to the edge of their territory towards the end of the conveyor. Therefore, the changes occur at the highest *y* value that the operator can reach in the case of the first two operators in Figure 9, Figure 10, Figure 11 and Figure 12 (e.g., around 2.7 m in the case of the first one). However, when the waste arrives at the third operator, it is already partially sorted; thus, there are fewer elements to be transferred, and the entropy, i.e., the mixedness of the waste, is close to zero afterward. As can be seen, the effect of this operator appears almost linear, which indicates that they are not overwhelmed, and their actions (corresponding to the piece of the largest *y*) take place equally distributed of the entire length of their territory.

It is also seen in Figure 12 that the largest information gain belongs to the third operator, despite the fact that he works the least, as the waste is already preselected by the first and second operators. This means that the actions of the third operator affect the mixedness of the waste (being near the pure state in this case) much more than those of the other two workers. This also implies that the curves should be investigated together, and not on their own.

The results above represent the steady-state operation of the system, as the input parameters were kept constant during the simulation. However, the created simulator is also applicable to execute dynamic analysis, which is one of our future research directions.

### 3.5. Application of the ROC Curve and Entropy to Determine Optimal Zone Borders

If the waste that leaves the conveyor belt is not completely separated, it is worth examining where the zone borders should be optimally defined when collecting the waste fractions at the end of the conveyor. For these investigations, the ROC curve (and Newton efficiency derived from it) as well as entropy seem appropriate tools. The separation threshold which is changed to obtain the ROC curve as declared in Section 2.3.1 corresponds to the zone borders here.

Of course, if the operators perform every action with the same imprecision (σ), then the optimal borders at the end of the belt will be the same as those defined for the operators. On the other hand, if the sorting strategy instructs each operator (that works with different imprecision) to focus on only one component class, i.e., put pieces in only one zone, then the optimal borders at the end will be different. As another example, it is also a consideration based on practical aspects that the operators (who can focus on all classes now) work with different imprecision in each zone. The closest zone to them can be considered with the lowest σ, as they can follow the trash piece with their hand until it is put down. However, if the target zone is the farthest from them, they may need to throw the piece there, which is a more inaccurate action with a higher σ.

**Figure 13:**
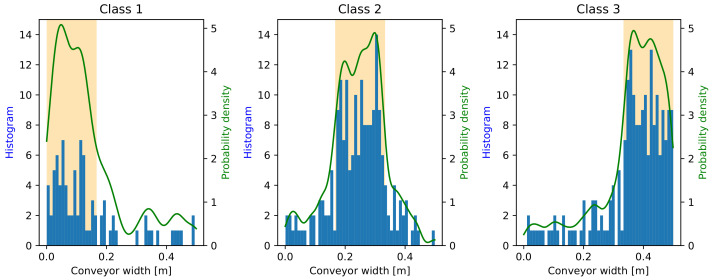
Distribution of the trash pieces from the three classes along the conveyor width (marked by *x* in Figure 4). The target zones corresponding to the classes are indicated by an orange background color. The histogram of the pieces can be seen in blue and the fitted probability distribution in green. The histograms and the probability densities are shown beside the same axis limits in all the cases of the three classes.

Using the considerations above, a case study is defined here, where there is one operator who works with different σ in the three zones: 0.05, 0.10, and 0.15 from the closest to the farthest zones, respectively. The action time was considered to be 1.5 s and the pieces were assumed to arrive at 2 $\frac{\mathrm{pcs}}{s}$ on the conveyor belt. The other parameters of the system (e.g., waste composition, conveyor size, and sorting strategy) were set the same as in Section 3.4, defined in Section 3.3. The simulator was run long enough to obtain a representative sample that left the conveyor, and the positions of these pieces along the conveyor width (*x* axis) were investigated by component classes, for which the distributions can be seen in Figure 13.

It can be seen in Figure 13, that most of the elements accumulated in their target zones in all classes. However, considering the probability densities (which are shown beside the same axis limits in the cases of the three classes), Class 1 shows a narrower peak than Classes 2 and 3, which corresponds to the higher precision of the operator in Zone 1.

**Figure 14:**
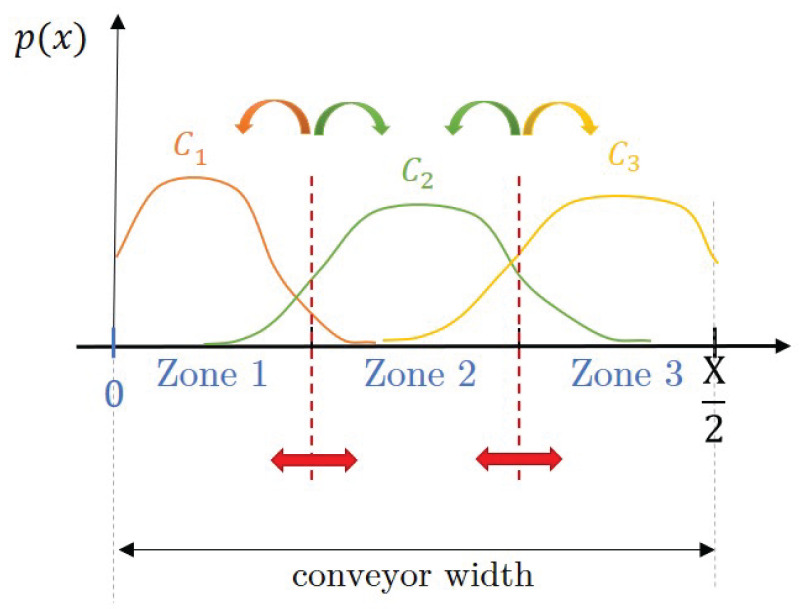
Connection between distribution of trash pieces from the classes along the conveyor width and zone borders. The valuable class—zone border pairs for which ROC curves are examined are marked by arrows in color corresponding the classes.

The ROC curves related to the defined system were gained by choosing a class as the valuable one, and defining the curve points by shifting one of its target zone borders and computing $TP_{rate}$ and $FP_{rate}$ for each case. Therefore, considering only the borders delimiting two zones with different classes matter, four ROC curves can be drawn, representing the effect of the border between Zone 1 and Zone 2 on the separation success of Class 1 (upper threshold) and Class 2 (lower threshold), and the effect of the one between Zone 2 and Zone 3 on the separation success of Class 2 (upper threshold) and Class 3 (lower threshold) as can be seen in Figure 14.

**Figure 15:**
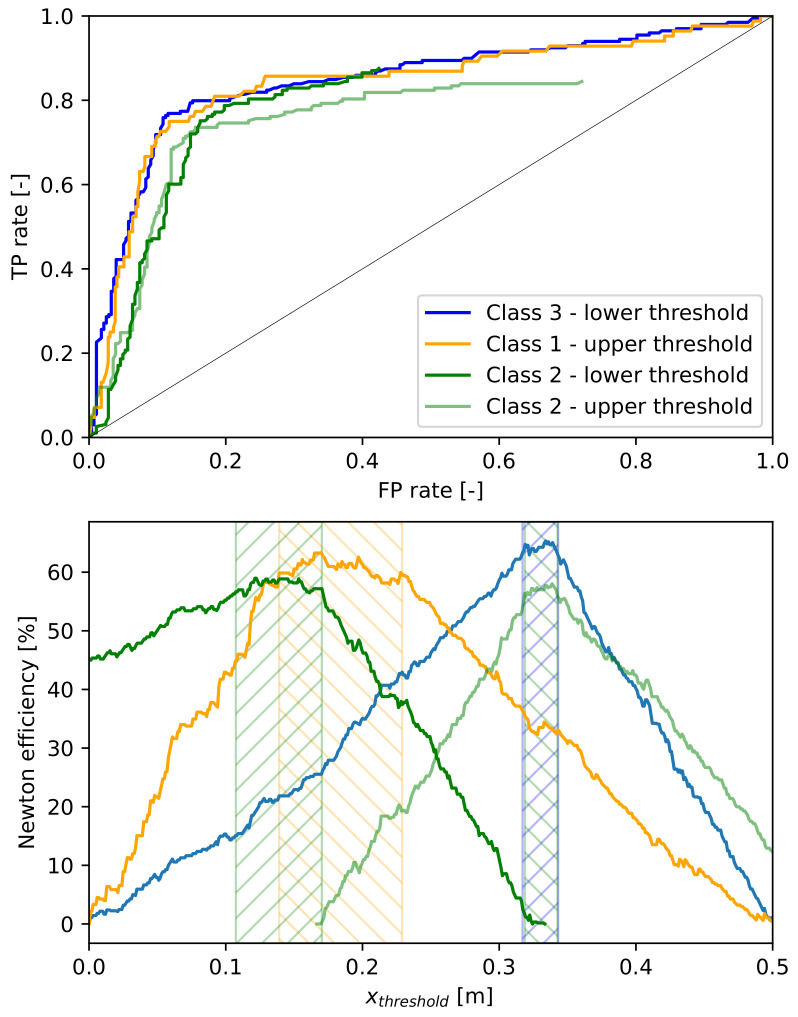
The ROC curves for the four class–border pairs defined above, and the Newton efficiencies related to the classes when the corresponding zone border is moved. The favorable intervals for zone borders based on the Newton efficiency curves are marked by a hatched background by the related color.

The gained ROC curves can be seen in Figure 15, together with the Newton efficiencies calculated from them by forming the difference of $TP_{rate}$ and $FP_{rate}$.

In Figure 15, the orange and dark green curves belong to the threshold between Zone 1 and Zone 2, and the blue and light green curves to the one between Zone 2 and Zone 3. It is seen that the latter ones are quite sharp with narrow hatched areas which cover each other; thus, they define the threshold explicitly. However, the orange and dark green curves show plateaus at their maximum. This means that the threshold values in the hatched zones give approximately the same separation performance regarding the corresponding class. For example, if Class 1 contains the valuable components that need to be recovered and the other classes do not matter, then each threshold value in the orange hatched zone (approximately between 0.14 and 0.22) will be appropriate. On the other hand, if Class 2 is considered the valuable class and the others are considered useless ones, the values of the dark green hatched zone (approximately between 0.11 and 0.17) give equally satisfying performance as a border between Zone 1 and Zone 2. If the recovery of both Class 1 and Class 2 is important, then the intersection of the orange and dark green hatched zones contains the optimal threshold values (approximately between 0.14 and 0.17).

The AUC values that belong to the four ROC curves are collected in Table 3.

As can be seen in Table 3, the output waste is largely separated (values around 80%), which is also seen in the ROC curves in Figure 15 that are far from the diagonal line. The curves belonging to Class 2 do not end at the (1,1) point because one of the borders of Zone 2 is fixed while the other is examined, and thus, a few trash pieces are inherently excluded, i.e., out of Zone 2. The AUC values of these curves are calculated by appending them with the (1,1) point so as to be able to compare them with the others. It is seen that they are a bit lower than those of Class 1 and Class 3 for the same reason.

**Figure 16:**
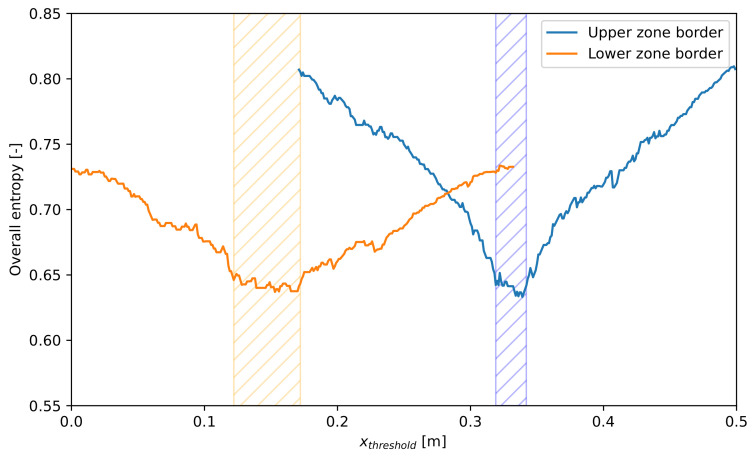
Overall entropy in relation to the location of zone borders. The favorable intervals for zone borders based on entropy are marked by a hatched background. The colors refer to the examined zone border.

If the separation of all component classes is equally important and examining them individually regarding the effect of the zone borders is not needed, the overall entropy is also applicable to determine the optimal thresholds. The entropy curves gained by fixing one of the zone borders and moving the other can be seen in Figure 16.

It can be seen in Figure 16 that the hatched zones have a position similar to that of Figure 15. Thereby, entropy is also an applicable metric to determine the optimal zone borders; however, the information about the sensitivity of separation success of individual classes to the zone borders is lost in this case.

In this section, it is shown how the entropy, ROC curves, and Newton efficiency derived from them can be applied to determine the optimal zone borders in a manual waste-sorting system that is described in Section 3.1. ROC curves are more favorable if the recovery of the classes individually is of interest, and entropy is preferable if the optimal solution is sought for considering all classes. The performance of other separation systems can also be evaluated similarly if a parameter can be found which can be changed and affects the separation performance. Different system types can also be compared according to their ROC curves.

## 4. Conclusions

This work aimed to involve classifier-based metrics in the evaluation of separation systems. The metrics and their corresponding interpretations in a discrete separation system were introduced, and their behaviors were analyzed in a case study of a manual waste-sorting system with stochastic nature. The created framework makes it possible to evaluate the system according to the user requirements by choosing the appropriate metrics, and to perform optimization tasks.

Separation systems can be considered classifiers: they divide a mixed input into fractions that are as pure as possible according to a particular property. As such, classifier-based metrics can be used to evaluate these systems. Some of them (e.g., information gain and entropy) highlight additional aspects of the system compared to traditional metrics, thus helping to obtain more complex views about it.

First, the investigated metrics and their representative meanings in the case of a separation system were defined. For example, the precision metric corresponds to the purity of the output streams, the entropy is applicable to characterize the mixedness of the material flow to be separated, and the information gain represents the effect of the operation units on it. A short outlook was also given on the connection between traditional efficiency measures and classifier-based metrics involving ROC curves, which are also applicable to determine the optimal cut-off values.

The behavior of the defined metrics was introduced in a case study of a manual waste-sorting system. A flexible simulator was created based on the stochastic model of the system, and the metrics were used to follow changes in the waste distribution along the conveyor belt and determine the effect of the operators. While the precision (representing the purity of the output streams or conveyor zones) and the recall (illustrating the recovery of class elements) increased monotonically as expected, the entropy in the zones (representing mixedness) did not show a monotonically decreasing tendency in certain cases, highlighting asymmetric waste composition. Therefore, involving this metric gave a more complex view about the operation of the system. Information gain was derived from the overall entropy to evaluate the operation units. ROC curves and entropy were applied to determine the optimal zone borders.

One of the main limitations of these metrics is that investigating them individually can sometimes be misleading. It was seen that the last operator who actively worked the least had the greatest effect on the mixedness of the waste, where it approached a zero-entropy state. However, examining them in relation to each other gives a complete picture about the system. Another limitation is that the person performing the system evaluation needs to know which aspect of the system is related to each metric. On the other hand, if the operation of a real system needs to be analyzed, the computation of some of the metrics requires a lot of measurement data. For example, in the case of entropy, the concentration of all components in the outputs has to be known. This problem can be eliminated by using state estimation techniques if not all the variables are measured but the system model is known.

In the future, we plan to involve other metrics used for the evaluation of manufacturing systems in the assessment of operation units. Additionally, we would like to use the defined metrics for the optimization of separation systems as well. For example, in the case of the introduced manual waste-sorting system, the sorting strategy of the operators could be optimized by performing sensitivity analyses on the system.

In conclusion, a more complex view about the separation system efficiency was gained by involving classifier-based metrics. This provides the chance to improve such systems by optimization (e.g., zone borders) and to define extended assessment structures considering different aspects all together using the corresponding performance-related metrics.

## Figures and Tables

**Table 1:** Parallelization of metrics and terms used for the evaluation of classifiers and separation systems, marking with X which part of the system the metrics characterize.

Classifiers	Separation	$F_{j}$	$C_{i}$	Whole System
precision (specificity)	purity of an output stream	x		
recall	recovery of a class		x	
accuracy	overall performance of the separation system/operators			x
entropy ($H_{j}$)	complexity/mixedness of an output stream	x		
overall entropy (*H*)	overall complexity of the system output			x
information gain (IG)	effect of the invested work on the complexity of the medium			x

**Table 2:** Composition of the waste feed in the simulation.

Component	Composition [%]
PET	9.7
LDPE	3.6
HDPE	2.3
Iron	2.7
Aluminum	1.0
Cardboard	13.4
Paper	23.0
Organic	44.3

**Table 3:** AUC values of the four ROC curves.

ROC Curve	AUC [-]
Class 1—upper threshold	0.8331
Class 2—lower threshold	0.8150
Class 2—upper threshold	0.7735
Class 3—lower threshold	0.8459

**Figure A1:**
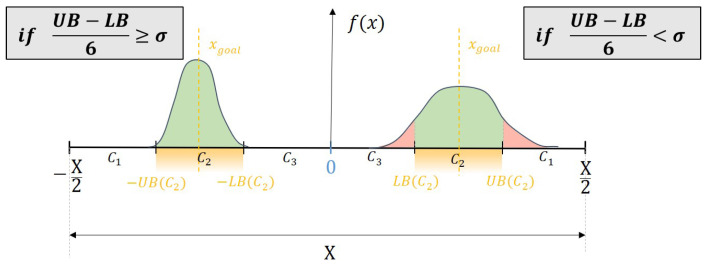
Expected position of a displaced trash piece that belongs to the $C_{2}$ class in the case of operators with high and low precision (i.e., small and large σ) on the cross-sectional diagram of the conveyor belt perpendicular to the *y* axis. f(x) denotes the probability density function of *x*. The appropriate *x* positions are represented by green and the incorrect ones by red.

## Data Availability

The original contributions presented in the study are included in the article, further inquiries can be directed to the corresponding authors.

## Appendix A. The Discrete Stochastic Model of the Waste-Sorting System

In this section, the created discretized model equations are described in detail, as well as the processes where stochasticity has to be considered.

### Appendix A.1. Main Model Equations

According to the Lagrangian approach, the (y,x) position of the trash pieces is wanted to be followed in time. These values are gathered in the *Y* and *X* matrices with dimensions ($N_{S}\times \mathrm{number}\;\mathrm{of}\;\mathrm{time}\;\mathrm{steps}$). Thereby, a column of these matrices gathers the *y* and *x* position values of all trash pieces that belong to a given timestamp.

The position of the trash pieces (marked by $W_{i}$, generally) is changed by two effects: the move of the conveyor and the operator actions. The conveyor moves along the *y* direction, and it is assumed that the operators change the position of the pieces only along the *x*-axis, while the position in the *y* direction remains the same. Thereby, the *y* position of the pieces can be calculated based on the model of the conveyor movement.

The conveyor moves with a constant speed *v* so the distance traveled per unit of time can be formalized:

Δy=vΔt,

which implies that the recursive formula of the *y* position of the trash pieces is:

$$Y\left(W_{i},t_{k}\right)=Y\left(W_{i},t_{k-1}\right)+v\cdot \left(t_{k}-t_{k-1}\right),$$

where $W_{i}$ refers to the trash pieces in general, $t_{k}$ is the current timestamp, $t_{k-1}$ is the previous one, and Y(i,j) denotes the element of the *i*th row and *j*th column of the *Y* matrix.

Similarly, the general recursive formula of the position along the *x* dimension can be defined considering the operator actions as:

$$X\left(W_{i},t_{k}\right)=X\left(W_{i},t_{k-1}\right)+\sum_{j=1}^{N_{O_{1}}}\Delta x\left(W_{i},t_{k}\right)\cdot \delta \left(W_{i},W_{O_{1,j}}\left(t_{k}\right)\right)\cdot \delta \left(0,t_{O_{1,j}}\left(t_{k}\right)\right)+\sum_{j=1}^{N_{O_{2}}}\Delta x\left(W_{i},t_{k}\right)\cdot \delta \left(W_{i},W_{O_{2,j}}\left(t_{k}\right)\right)\cdot \delta \left(0,t_{O_{2,j}}\left(t_{k}\right)\right),$$

where all the operator actions are considered. $W_{O_{1,j}}\left(t_{k}\right)$ and $W_{O_{2,j}}\left(t_{k}\right)$ refer to the trash pieces chosen to move at time $t_{k}$ by the *j*th $O_{1}$- or $O_{2}$-type operator, respectively. δ(·) denotes the generalized form of the Kronecker delta function, which gives 1 when its arguments are identical or equal, and 0 otherwise. $t_{O_{1,j}}\left(t_{k}\right)$ and $t_{O_{2,j}}\left(t_{k}\right)$ variables are time counters belonging to the operators as will be determined in Equation (A2), which aim to define whether the operators are busy or not at time $t_{k}$. For example, $t_{O_{1,j}}\left(t_{k}\right)$ takes 0 if the *j*th $O_{1}$ operator is free, until they can choose an element to work with. $\Delta x(W_{i},t_{k})$ represents the displacement distance of the $W_{i}$ trash piece at time $t_{k}$, which is:

$$\Delta x\left(W_{i},t_{k}\right)=x_{new}-x\left(W_{i},t_{k-1}\right),$$

for the pieces to be moved, where $x_{new}∼N\left(x_{goal},\sigma \right)$. $x_{goal}$ refers to the target position of the trash piece, which is the middle of the target zone as follows:

(A1) $$x_{goal}\left(W_{i}\right)=\left\{\begin{array}{cc} \frac{LB\left(C\left(W_{i}\right)\right)+UB\left(C\left(W_{i}\right)\right)}{2} & \mathrm{if}\;W_{i}\in {\left\{W_{O_{1,j}}\right\}}_{j=1}^{N_{O_{1}}}\\ -\frac{LB\left(C\left(W_{i}\right)\right)+UB\left(C\left(W_{i}\right)\right)}{2} & \mathrm{if}\;W_{i}\in {\left\{W_{O_{2,j}}\right\}}_{j=1}^{N_{O_{2}}}, \end{array}\right.$$

where $C(W_{i})$ represents the class which the $W_{i}$ piece belongs to, and LB and UB are the lower and upper boundaries of the zone on the conveyor belt as a positive number, respectively. The standard deviation denoted by σ symbolizes the imprecision of the operation actions that will be discussed in more detail in Appendix A.2.

After clarifying the principles of defining the target position ($x_{new}$) of the trash pieces, the only remaining question is how to choose trash pieces for the operators to move. It is assumed that it takes $\Delta t_{O}$ time for an operator to move a trash piece, and that every operator deals with only one piece at a time. Thereby, new trash pieces are only chosen for the free operators, and if a trash piece is chosen (e.g., $W_{O_{1,j}}$ is defined) at time $t_{k}$, then

$$W_{O_{1,j}}\left(t_{k}\right)=W_{O_{1,j}}\left(t_{k+1}\right)=\;...\;=W_{O_{1,j}}\left(t_{k}+\Delta t_{O}\right),$$

and a timer variable belonging to it (let it be denoted by $t_{O_{1,j}}$ for $W_{O_{1,j}}$) starts to count time and sets back to 0 if the action is done:

(A2) $$t_{O_{1,j}}\left(t_{k}\right)=\left\{\begin{array}{cc} t_{O_{1,j}}\left(t_{k-1}\right)+\left(t_{k}-t_{k-1}\right) & \mathrm{if}\;t_{O_{1,j}}\left(t_{k-1}\right)\leq \Delta t_{O}\;\;\mathrm{and}\;\;W_{O_{1,j}}\left(t_{k-1}\right)\neq ⌀\\ 0 & \mathrm{else}. \end{array}\right.$$

To choose a new trash piece to move for an operator, four aspects have to be considered:

1. The operator has to be free to receive a new task.
2. The new piece cannot be under the treatment of another operator.
3. The operator has to be able to reach the piece considering both the *x* and *y* directions.
4. The piece chosen to move has to be in the wrong zone regarding its class.

These aspects are summarized in Equation (A3) for the case of the *j*th $O_{1}$-type operator:

(A3) $$W_{O_{1,j}}\left(t_{k}\right){|}_{W_{O_{1,j}}\left(t_{k}\right)=⌀}=\underset{W_{i}}{\mathrm{argmax}}\left(y\left(W_{i},t_{k}\right)\cdot e\left(W_{i},t_{k}\right)\right)\;\left|\begin{array}{c} W_{i}\notin {\left\{W_{O_{1,m}}\left(t_{k}\right),W_{O_{2,n}}\left(t_{k}\right)\right\}}_{m=1,...,N_{O_{1}},n=1,...,N_{O_{2}}}\\ y_{O_{1,j}}-\Delta y_{O}\leq y\left(W_{i},t_{k}\right)\leq y_{O_{1,j}}+\Delta y_{O}\\ 0\leq x\left(W_{i},t_{k}\right)\leq \frac{X}{2}\\ (\mathrm{or}\;-\frac{X}{2}\leq x\left(W_{i},t_{k}\right)\leq 0\;\mathrm{for}\;\mathrm{an}\;O_{2}\;\mathrm{type}\;\mathrm{operator}), \end{array}\right.$$

where

$$e\left(W_{i},t_{k}\right)=\left\{\begin{array}{cc} 0 & \mathrm{if}\;LB\left(C\left(W_{i}\right)\right)\leq \left|x\left(W_{i},t_{k}\right)\right|\leq UB\left(C\left(W_{i}\right)\right)\\ 1 & \mathrm{otherwise}, \end{array}\right.$$

representing if the $W_{i}$ piece is in the right zone or not.

### Appendix A.2. Incorporation of Stochastic Effects into the Model

In the system outlined in the previous sections, different stochastic phenomena takes place. The two main representatives of these are related to the modeling of the arriving trash pieces and the precision of the operator actions.

The trash pieces are assumed to arrive one after another at the beginning of the conveyor belt exactly on the y=0 line. There are three aspects of the new pieces in which randomness appears:

- Position along the *x* axis;
- Component type;
- Arrival time.

Regarding their initial position along the *x* axis, it is assumed that they are uniformly distributed on the full width of the conveyor; thereby, a value is drawn from the $\left[-\frac{X}{2};\frac{X}{2}\right]$ interval randomly. The component type of the new piece is chosen from the $\left\{c_{1},c_{2},...,c_{N_{comp}}\right\}$ components, each with a probability related to their proportion in the feed.

For modeling the arrivals of the trash pieces, queuing theory is involved. The “birth” of the pieces is considered a Poisson process in this research, where the interarrival times are independent from each other and follow an exponential distribution with the λ rate parameter, which is the reciprocal of the mean of interarrival times [29].

When an operator moves a trash piece, the target position ($x_{goal}$) is the middle of the zones belonging to its class as defined by Equation (A1). However, the action will never be completely precise, which means that the displaced piece will not exactly reach the target position every time. In cases of high imprecision (i.e., a large value of σ), the piece may not even arrive at the target zone as shown in Figure A1.

Figure A1 illustrates the difference between the performance of operators with high precision (on the left with small σ) and low precision (on the right with large σ). The value of σ can be qualified using the principles of the so-called process capability analysis [38]. Process capability ($C_{p}$) in the present system can be defined as:

$$C_{p}=\frac{UB-LB}{6\sigma }.$$

$C_{p}\geq 1$ means that 99.7% of the system outputs are within the specification limits (LB and UB), i.e., almost all of the trash pieces will reach the right zone. According to these principles, a threshold for σ (as $\frac{UB-LB}{6}$) can be defined, below which the operator actions are completely precise, and beyond which there will be wrong displacements as well. This is also presented in Figure A1. Operator precision represented by σ is considered a fixed value in this work, and thus only the width of the zones can be adjusted to it.

In addition to the above-mentioned, there are other factors in the system that may possess uncertainty but were considered invariant in the experiments introduced in this paper. For example, the load and composition of feed waste can be volatile in a real-world problem, and the operator action time can have a randomness as well. The σ value representing the imprecision of the operators can also vary, e.g., depending on the workload.
